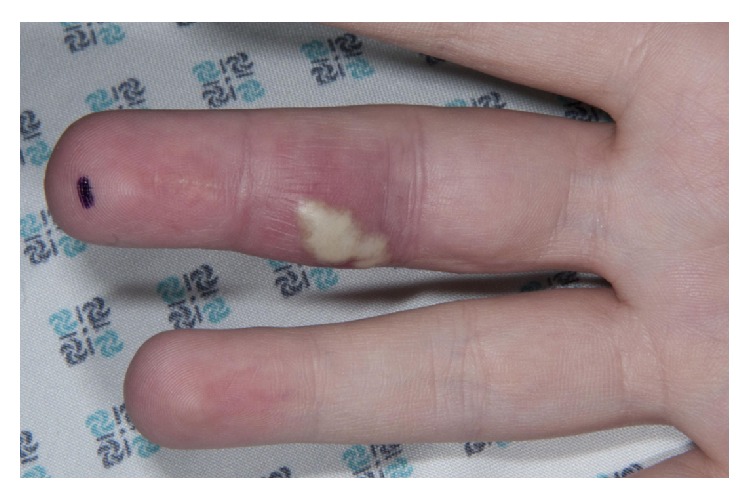# A Case Report of Herpetic Whitlow with Positive Kanavel's Cardinal Signs: A Diagnostic and Treatment Difficulty

**DOI:** 10.1155/2014/906487

**Published:** 2014-11-04

**Authors:** Milos Brkljac, Samer Bitar, Zafar Naqui

**Affiliations:** Salford Royal Foundation Trust, Manchester, UK

## Abstract

Herpetic whitlow is an acute viral infection of the hand caused by either herpes simplex virus (HSV) 1 or 2. Its characteristic findings are significant pain and erythema with overlying nonpurulent vesicles. The differential diagnosis includes flexor tenosynovitis. We present a case of recurrent infection of the middle finger in an immunocompetent 19-year-old girl. Multiple painful pustules with tracking cellulitis were partially treated by oral antibiotics. A recurrence with positive Kanavel's signs suggested flexor tenosynovitis at seven months. Her symptoms improved transiently following emergent surgical open flexor sheath exploration and washout however, she required two further washouts; at eleven and thirteen months to improve symptoms. Viral cultures were obtained from the third washout as HSV infection was disclosed from further history taking. These were positive for HSV2. Treatment with acyclovir at thirteen months after presentation led to a complete resolution of her symptoms with no further recurrences to date. This rare case highlights the similarity in presentation between flexor sheath infection and herpetic whitlow which can lead to diagnostic confusion and mismanagement. We emphasise the importance of careful past medical history taking as well as considering herpetic whitlow as a differential diagnosis despite the presence of strongly positive Kanavel's signs.

## 1. Introduction

Herpetic whitlow is an acute viral infection of the hand caused by herpes simplex virus (HSV 1/HSV 2). Its characteristic findings are significant pain and localised erythema followed by development of small nonpurulent vesicles. Differential diagnoses include flexor tenosynovitis, bacterial felon, and paronychia [1]. The similarity in presentation between flexor sheath infection and herpetic whitlow can lead to diagnostic confusion and mismanagement. This can be aggravated further when Kanavel's cardinal signs are strongly positive. We present our case of recurrent finger infection in a 19-year-old female and we emphasise the importance of careful past medical history taking which can help in reaching an accurate diagnosis as well as considering herpetic whitlow as a differential diagnosis despite the presence of Kanavel's signs.

## 2. Case Report

A 19-year-old girl presented to her General Practitioner with a painful erythematous middle finger with tracking erythema up the arm. She was started on oral antibiotics (Flucloxacillin) for presumed cellulitis. She was and remained systemically well; however, she required hospital admission three days later when infection failed to respond to antibiotics. The finger was noted to be more swollen and exquisitely painful with reduced range of movement. Clinical examination revealed small, firm, circular, and painful pustules along with erythema spreading to the level of axilla accompanied by regional lymphadenopathy. Kanavel's cardinal signs were all positive including intense pain on attempting to extend her partially flexed finger, flexion posture, uniform swelling, and percussion tenderness. The decision was made to proceed with surgical open flexor sheath exploration and washout. Clear fluid was noted and specimens were sent for standard culture and sensitivity. These came back negative; however, symptoms did resolve following washout.

Seven months later, the patient presented with similar symptoms, however, this time, with more localised symptoms to the finger only. A repeat flexor sheath washout was undertaken which resulted in symptomatic improvement. A similar third presentation four months following the second presentation was treated in the same fashion. A biopsy was obtained on this occasion with cultures and sensitivities for atypical organisms; these were negative.

**Figure 1:**
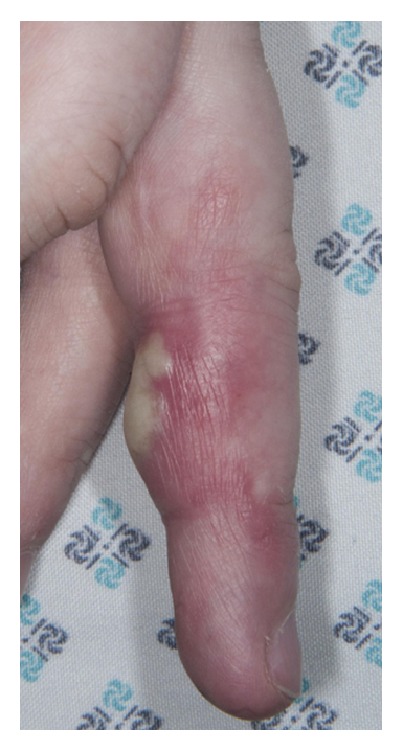


**Figure 2:**
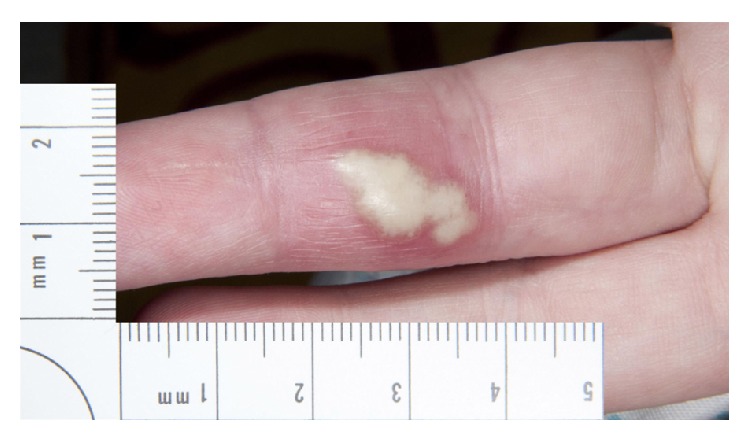


**Figure 3:**
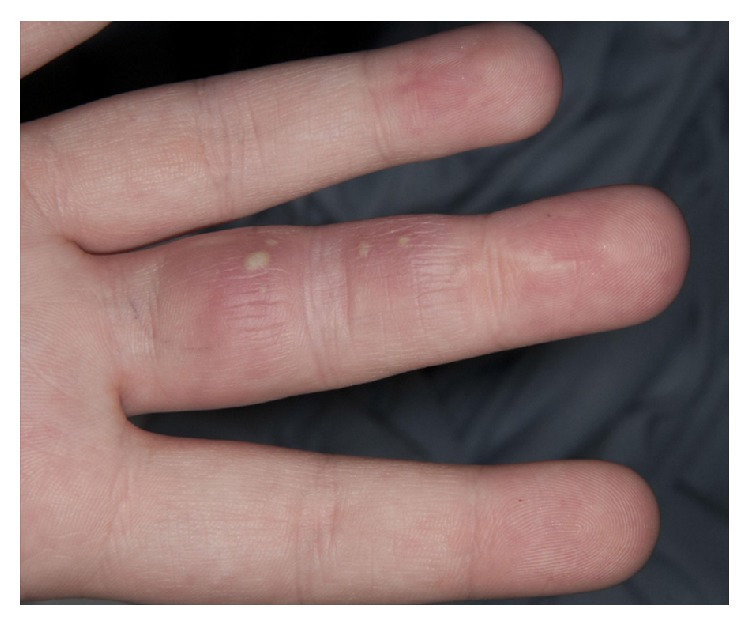


Two months after the third washout, she was rereferred to the hand service by her General Practitioner with a painful swollen middle finger partially covered with multiple small pustules as seen in Figures 1, 2, 3 and 4. However, Kanavel's signs were not present this time. A more detailed history revealed a pervious herpes simplex virus (HSV) infection as a child. The pustules were surgically deroofed and thick fluid was drained in theatre. Samples of tissue and fluid were sent for microbiological analysis including a swab in a viral transport medium. HSV type 2 was confirmed following a positive culture and polymerase chain reaction (PCR). The diagnosis of herpetic whitlow was established. She received 200 mg of Acyclovir five times a day for seven days and, at thirteen months after presentation, this led to complete resolution of her symptoms with no further recurrences up to the time of writing.

## 3. Discussion

The first published report of herpetic whitlow of the finger in adults was in 1909 by Adamson [2]. The classical vesicles tend to arise after a few days of skin irritation or minor trauma and may include a prodromal period of flu-like symptoms. Herpetic whitlow is a clinical diagnosis and its treatment differs greatly from other common hand infections; thus, special attention must be paid to examination findings and history alike.

Vesicles are usually clear or pale yellow, have a base which is erythematous, and can coalesce into a single vesicle [3]. Regional lymphadenopathy may accompany these findings; however, systemic symptoms are rare [4].

The flexor sheath infection should be considered in the differential diagnosis of a painful swollen digit and Kanavel's signs [5] are in most cases a useful tool. The absence of these signs and the presence of vesicles aid the diagnosis and guide further management of herpetic whitlow. However, in even rarer cases, as in our case, the presence of these signs does not preclude the diagnosis of herpetic whitlow which should be considered in recurrent refractory cases. We therefore feel that herpetic whitlow can present in atypical way and can very rarely largely mimic flexor sheath infection presenting with positive Kanavel's signs making the correct diagnosis more challenging. We emphasise the importance of detailed past medical history, careful clinical examination, and careful assessment of Kanavel's signs as well as early consideration of viral cultures in recurrent cases.

HSV infection can be confirmed using Tzanck test, viral culture, or PCR. Often, the condition is self-limiting and will resolve in a few weeks [6]. Treatment using antiretrovirals such as acyclovir should be initiated within 48 hours and can be effective in recurrent infections if started during the prodrome.

## 4. Conclusion

We understand that herpetic whitlow is rare and has always been a differential diagnosis for flexor sheath infection; however, in most cases, the absence of Kanavel's signs and the presence of vesicles aid the diagnosis. Herpetic whitlow can present in atypical way and can mimic flexor sheath infection considerably, presenting with Kanavel's signs, thus making the diagnosis more challenging. We emphasise the importance of detailed past medical history, careful clinical examination, and vigilant assessment of Kanavel's signs as well as early consideration of viral cultures in recurrent cases.

## Figures and Tables

**Figure 4:**